# Nonlinear Demodulation and Channel Coding in EBPSK Scheme

**DOI:** 10.1100/2012/180469

**Published:** 2012-11-20

**Authors:** Xianqing Chen, Lenan Wu

**Affiliations:** School of Information Science and Engineering, University of Southeast, 2 Sipailou, Nanjing 210096, China

## Abstract

The extended binary phase shift keying (EBPSK) is an efficient modulation technique, and a special impacting filter (SIF) is used in its demodulator to improve the bit error rate (BER) performance. However, the conventional threshold decision cannot achieve the optimum performance, and the SIF brings more difficulty in obtaining the posterior probability for LDPC decoding. In this paper, we concentrate not only on reducing the BER of demodulation, but also on providing accurate posterior probability estimates (PPEs). A new approach for the nonlinear demodulation based on the support vector machine (SVM) classifier is introduced. The SVM method which selects only a few sampling points from the filter output was used for getting PPEs. The simulation results show that the accurate posterior probability can be obtained with this method and the BER performance can be improved significantly by applying LDPC codes. Moreover, we analyzed the effect of getting the posterior probability with different methods and different sampling rates. We show that there are more advantages of the SVM method under bad condition and it is less sensitive to the sampling rate than other methods. Thus, SVM is an effective method for EBPSK demodulation and getting posterior probability for LDPC decoding.

## 1. Introduction

Nowadays, wireless communication is playing a very important role in our daily life. The growing demands on wireless multimedia services and products lead to increasing needs for radio spectrum and data rates. Thereby, the research on modulations with high bandwidth efficiency is on focus [1]. In order to satisfy the higher and higher demand for communication systems, an extended binary phase shift keying (EBPSK) system with very high spectra efficiency is introduced in [2]. A special impacting filter (SIF) which can produce high impact at the phase jumping point, narrow in bandwidth, and great improvement in SNR, was applied at the demodulator [3]. Therefore, a simple amplitude detector followed would perform the demodulation of EBPSK signals [4]. However, the conventional threshold decision may not be best to achieve the optimum performance, and the SIF used in EBPSK demodulator brings more difficulty in obtaining posterior probability for low-density parity check (LDPC) codes decoding. A simple and general bit metric generation method is proposed by Hyun and Yoon [5] for the soft information to initial channel decoding. We modify the scheme to suit our system and the method is referred to as MHY in this paper. Meanwhile, nonlinear detectors are specifically designed to get the optimum performance of a blind multiuser detector [6, 7] and nonlinear channel equalization [8–10] and providing accurate posterior probability estimates (PPEs) for LDPC decoding [11, 12]. All results have shown that a nonlinear demodulator performs similar to an optimum receiver. One of the goals of this paper is the analysis of nonlinear demodulation with the channel decoder. We make use of the fact that the demodulator performance should not only be measured by low BER, but also in its ability to provide accurate PPEs that can be exploited by a soft-input channel decoder to achieve capacity. In this paper, we will introduce a nonlinear demodulation technique called the support vector machine (SVM) classifier [13]. The design approach is completely novel, where we select only a few samples of the SIF output for SVM training and testing at intermediate frequency (IF) without downconversion. We propose to measure the performance of this demodulator after an LDPC channel decoder, and the ability of SVM to provide accurate posterior probability predictions boosts the demodulator performance compared to the MHY method.

The rest of the paper is organized as follows.  Section 2 is devoted to introducing SVM. We present the receiver scheme in Section 3 and briefly describe the EBPSK modulation and LDPC decoding. In Section 4, we include illustrative experiments to compare the performance of the proposed demodulators. We conclude in Section 5 with some final comments.

## 2. Support Vector Machine

The SVM is a classifier introduced by Cortes and Vapnik [14], which can realize the same performance as the so-called artificial neural networks (ANNs) for classification. Generally, ANN has the problem of a local minimum. On the other hand, the SVM is mathematically transparent and can provide global and unique solutions.

### 2.1. Binary Classification of SVM

For the binary classification problem, the training set consists of vectors from the pattern space **x**_*i*_ ∈ **R** ^*n*^, *i* = 1,  2,…,  *L* and to each vector a classification *y*_*i*_ ∈ {1, −1}. During the initial training stage, a decision function is constructed via

(1) $$\begin{array}{c} f\left(x\right)=\sum_{i=1}^{L}\alpha_{i}y_{i}K\left(x,x_{i}\right)+b, \end{array}$$

where *α*_*i*_ is a Lagrangian constant, *K*(**x**, **x**_*i*_) = Ψ(**x**_*i*_)^*T*^ · Ψ(**x**) is a kernel function, Ψ(**x**) maps the training data vector **x**_*i*_ into the high-dimensional feature space, and *b* is a bias term.

Define a coefficient vector **w**, such that

(2) $$\begin{array}{c} w=\sum_{i=1}^{L}\alpha_{i}y_{i}\Psi \left(x_{i}\right), \end{array}$$

then the training is completed by solving the following optimization problem:

(3) minw∈H, b∈R, ξ∈RL12||w||2+C∑i=1Lξiyi((w·xi)+b)≥1−ξi,ξi≥0,  i=1, 2,…, L,

where *C* is the tradeoff parameter between the training error and the margin of the decision function, and *ξ*_*i*_ is a slack variable to compensate for any nonlinearly separable training points.

In this paper, the SVM demodulator uses two types of kernel functions to compare the performance with each other. The first is the simplest linear kernel, shown as

(4) $$\begin{array}{c} K\left(x_{i},x_{j}\right)=x_{i}^{T}x_{j}. \end{array}$$

The second is a more popular radial basis function (RBF) kernel, shown as

(5) $$\begin{array}{c} K\left(x_{i},x_{j}\right)=\exp\left(-\gamma {||x_{i}-x_{j}||}^{2}\right),\;\gamma >0, \end{array}$$

where *γ* controls the width of the function.

### 2.2. Complexity Analysis

The complexity of training an SVM for binary classification is *O*(*n* ^2^), using the sequential minimal optimization [15], and Platt's method adds a computational complexity of *O*(*n* ^2^). However, the SVM demodulator should be analyzed for the testing stage only because the training time is very small compared with the actual testing time. The main focus thus becomes analyzing the complexity required for the computing decision function in (1), which is using the simplest kernel. This issue will be discussed in detail later. A great amount of complexity can be reduced further in (1) if the expression is simplified as follows:

(6) f(x)=∑i=1LαiyiK(x,xi)+b=∑i=1Lαiyi(xiTx)+b=[∑j=1Nyjαj(∑i=1nxj,ixi)]+b=∑i=1nxi(∑j=1Nyiαixj,i)+b=∑i=1nAixi+b,

where *N* is the number of support vectors, and the constants *A*_*i*_ = ∑_*j*=1_^*N*^ *y*_*i*_ *α*_*i*_ *x*_*j*,*i*_ and *b* can be precomputed before the testing stage to save the computation time. Therefore, the complexity of the SVM demodulator is *O*(*n*).

### 2.3. Probabilistic Outputs of SVM

Instead of predicting the label, many applications require a posterior class probability *P*(*y* = 1 | *x*). The transformation of SVM output into posterior probabilities has been proposed by Platt in [16]. Platt's method squashes the SVM soft output through a trained sigmoid function to predict posterior probabilities:

(7) $$\begin{array}{c} p\left(y=1\;|\;x\right)\approx P_{A,B}\left(f\right)=\frac{1}{1+\exp\left(Af+B\right)}, \end{array}$$

where *f* = *f*(**x**), let each *f*_*i*_ be an estimate of *f*(**x**_*i*_). The best parameter setting z* = (A*, B*) is determined by solving the following regularized maximum likelihood problem:

(8) $$\begin{array}{c} \underset{z=\left(A,B\right)}{\min}F\left(z\right)=-\sum_{i=1}^{l}\left(t_{i}\log\left(p_{i}\right)+\left(1-t_{i}\right)\log\left(1-p_{i}\right)\right), \end{array}$$

where *p*_*i*_ = *P*_*A*,*B*_(*f*_*i*_), *t*_*i*_ = (*y*_*i*_ + 1)/2.

Furthermore, log and exp could easily cause an overflow, if *Af*_*i*_ + *B* is large, exp⁡⁡(*Af*_*i*_ + *B*) → *∞* and 1 − *p*_*i*_ = 1 − 1/(1 + exp⁡(*Af*_*i*_ + *B*)) is a “catastrophic cancellation” when *p*_*i*_ is close to one. The problem can usually be resolved by reformulation [17]:

(9) $$\begin{array}{c} -\left(t_{i}\log p_{i}+\left(1-t_{i}\right)\log\left(1-p_{i}\right)\right) \end{array}$$

(10) $$\begin{array}{c} \;\;=\left(t_{i}-1\right)\left(Af_{i}+B\right)+\log\left(1+\exp\left(Af_{i}+B\right)\right) \end{array}$$

(11) $$\begin{array}{c} \;\;=t_{i}\left(Af_{i}+B\right)+\log\left(1+\exp\left(-Af_{i}-B\right)\right). \end{array}$$

If *Af*_*i*_ + *B* ≥ 0 then use (11), else use (10). Then (7) can be rewritten as follows:

(12) $$\begin{array}{c} p\left(y=1\;|\;x\right)\approx \{\begin{array}{cc} \frac{1}{1+\exp\left(Af+B\right)}, & Af+B<0,\\ \frac{\exp\left(-Af-B\right)}{1+\exp\left(-Af-B\right)}, & Af+B\geq 0. \end{array} \end{array}$$

From (12), we can see that SVM does not provide PPE and its output needs to be transformed, before it can be interpreted as posterior probabilities; therefore, the posterior probability is an approximate one.

## 3. Communication System

### 3.1. EBPSK Modulation

EBPSK is a modulation method with high frequency spectra efficiency, which is defined as follows:

(13) $$\begin{array}{c} f_{0}\left(t\right)=A\sin2\pi f_{c}t,\;0\leq t<T,\\ f_{1}\left(t\right)=\{\begin{array}{cc} B\sin\left(2\pi f_{c}t+\theta \right), & 0\leq t<\tau ,\;0\leq \theta \leq \pi ,\\ A\sin\left(2\pi f_{c}t\right), & \tau \leq t<T, \end{array} \end{array}$$

where*f*_0_ and *f*_1_ are modulation waveforms corresponding to bit “0” and bit “1,” respectively, *T* = *N*/*f*_*c*_ is the bit duration, *τ* = *K*/*f*_*c*_ is the phase modulation duration, and *θ* is the modulating angle. Obviously, if *τ* = *T* and *θ* = *π*, (13) degenerates to the classical binary phase shift keying (BPSK) modulation.

### 3.2. LDPC Decoding

LDPC codes can be decoded by an iterative message-passing (MP) algorithm which passes messages between the variable nodes and check nodes iteratively. If the messages passed along the edges are probabilities, then the algorithm is also called belief propagation (BP) decoding, which is the optimal if there are no cycles or cycles are ignored. Moreover, with BP decoding, complicated calculations are distributed among simple node processors, and after several iterations, the solution of the global problem is available. The steps of BP decoding are as follows.

(1) Initialization: *p*_*n*_^0^(*x*) = *q*_*nm*_^0^ = *p*(*x*_*n*_ = *x* | *y*_*n*_), where *p*(*x*_*n*_ = *x* | *y*_*n*_) is the soft information of channel outputs.
(2) Horizontal step: the MAP output from *c*_*m*_ to *v*_*n*_:
  (14) $$\begin{array}{c} r_{mn}^{k}\left(0\right)=p\left(v_{n}=0\;|\;c_{m}=0,y_{i\in B(m)\setminus n}\right),\\ r_{mn}^{k}\left(0\right)=\frac{1}{2}+\frac{1}{2}\prod_{i\in B\left(m\right)\setminus n}\left(1-2q_{im}^{k}\left(1\right)\right),\\ r_{mn}^{k}\left(1\right)=1-r_{mn}^{k}\left(0\right). \end{array}$$
(3) Vertical step: updating the message from *v*_*n*_ to *c*_*m*_:
  (15) $$\begin{array}{c} q_{nm}^{k+1}\left(0\right)=\theta p_{n}^{0}\left(0\right)\prod_{j\in A(n)\setminus m}r_{jn}\left(0\right),\\ q_{nm}^{k+1}\left(1\right)=\theta p_{n}^{0}\left(1\right)\prod_{j\in A(n)\setminus m}r_{jn}\left(1\right),\;\;\theta \;\text{is}\;\text{chosen}\;\text{to}\;\text{ensure}\\ q_{nm}^{k+1}\left(0\right)+q_{nm}^{k+1}\left(1\right)=1,\;\;\text{Compute}\;\;p_{n}^{k}\left(x\right),\\ p_{n}^{k+1}\left(0\right)=\theta p_{n}^{0}\left(0\right)\prod_{j\in A(n)}r_{jn}\left(0\right),\\ p_{n}^{k+1}\left(1\right)=\theta p_{n}^{0}\left(1\right)\prod_{j\in A(n)}r_{jn}\left(1\right). \end{array}$$
(4) Tentative output:
  (16) $$\begin{array}{c} v_{n}^{k+1}=\{\begin{array}{cc} 1, & p_{n}^{k+1}\left(1\right)\geq 0.5,\\ 0, & p_{n}^{k+1}\left(1\right)<0.5, \end{array} \end{array}$$
  if all parity check equations are satisfied or the maximum iteration number is reached, stop iteration, else return to Step (2).

In this paper, we focus on the initialization step for the posterior probabilities obtained by the nonlinear demodulator.

### 3.3. System Model

**Figure 1:**
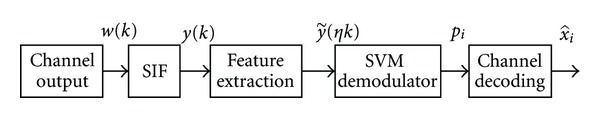
The block diagram of EBPSK receiver.

Figure 1 shows the receiver of EBPSK system. Suppose the system is synchronized, the signal of the channel output can be expressed as *w*(*k*) = *z*(*k*) + *n*(*k*), where *n*(*k*) is Gaussian white noise with zero mean. Input *w*(*k*) into a SIF, and then the output signal can be expressed as *y*(*k*) = *w*(*k*)∗*h*(*k*), where *h*(*k*) is the impulse response of SIF. In order to reduce the demodulation complexity, we select a few sample points as the features for SVM training and testing. Then, using the decision function (1), we can get the binary output as follows:

(17) $$\begin{array}{c} \mathrm{sign}\left(f\left(\tilde{y}\left(\eta k\right)\right)\right)=\mathrm{sign}\left(\sum_{i=1}^{l}\alpha_{i}c_{i}K\left({\tilde{y}}_{i}\left(\eta k\right),\tilde{y}\left(\eta k\right)\right)+b\right). \end{array}$$

Then, we can get the posterior probability $p({\tilde{x}}_{i}=1|{\tilde{y}}_{i}(\eta k))$ and $p\left({\tilde{x}}_{i}=0|{\tilde{y}}_{i}\left(\eta k\right)\right)=1-p({\tilde{x}}_{i}=1|{\tilde{y}}_{i}(\eta k))$ through (12): finally, we use *p*(*x*_*i*_ = *x* | *y*_*i*_) to initiate the LDPC decoder.

## 4. Simulation Results and Discussions

In this section, we illustrate the performance of the proposed SVM demodulation and its soft output for LDPC decoding. Unless specified otherwise, all simulations assume that the system had 3000 random symbols for training and the reported BER is computed using 10^5^ symbols and we average the results over 1000 independent trials with random training and test data. We choose *K* = 2, *N* = 20, *A* = *B* = 1, *θ* = *π* as the parameters of EBPSK modulation. LDPC codes are also applied to measure the BER performance of the communication system and the accurate posterior probability obtained by the SVM method. During simulations, we use a 1*⁄*2 rate regular LDPC code with 1000 bits per codeword and 3 ones per column. The whole system was simulated under MATLAB.

### 4.1. Kernel Selection and Demodulation

In this subsection, the performance of the SVM demodulator, using the kernel functions (4) and (5), introduced in Section 2, is compared. For the RBF kernel, a 10-fold cross-validation sweep from the training samples was used to find the optimum parameters of *C* and *γ*. A similar search was conducted for the linear kernel, but it only has the *C* parameter to adjust.  Table 1 summaries the optimum SVM model obtained after the parameter search.

The linear kernel has less support vectors than the RBF one; therefore, it has a less computational complexity and thus would perform faster. In order to compare the BER performance fairly, both kernels used by the SVM receiver were classifying exactly the same received signals.

**Figure 2:**
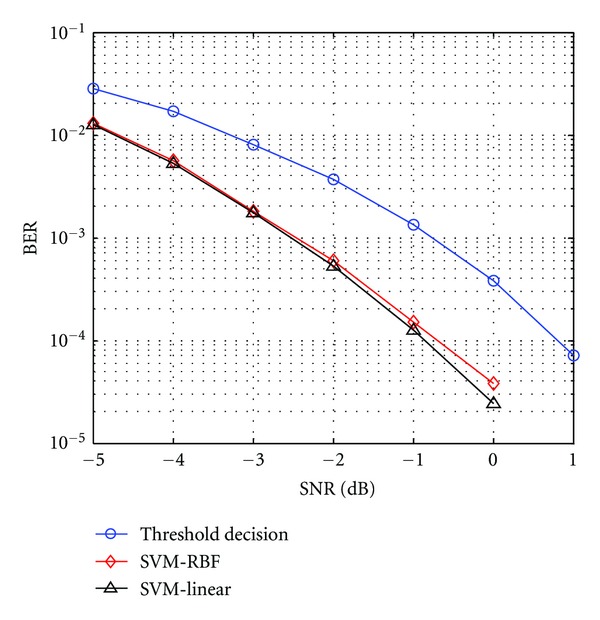
Demodulation with SVM-RBF, SVM-linear, and threshold decision.

Figure 2 shows the BER performance of the SVM demodulator when employing different kernels; also, the performance of conventional threshold decision is analyzed. Evidently, the linear kernel, though much simpler, has slightly better performance than the RBF kernel. Moreover, the SNR gain between the SVM method and the threshold decision is around 1.8 dB; therefore, a linear SVM is chosen for the task. Training on a “worse-case” scenario works well (SNR = −7 dB in this case), proving that the SVM receiver needs not frequently retraining in different SNRs.

### 4.2. Kernel Optimization

**Figure 3:**
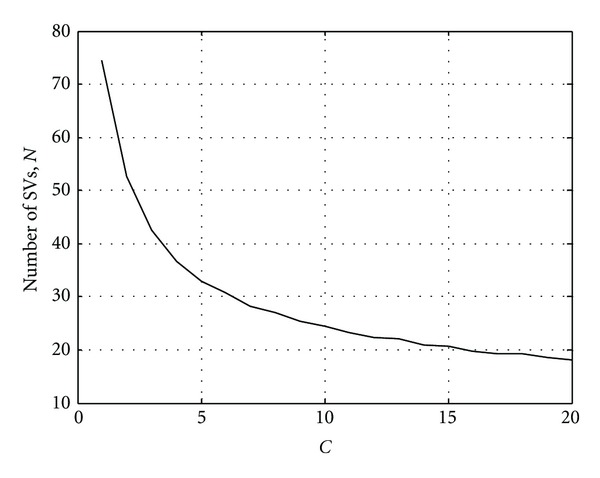
Number of support vectors from the SVM model for different *C* parameters, *n* = 5.

**Figure 4:**
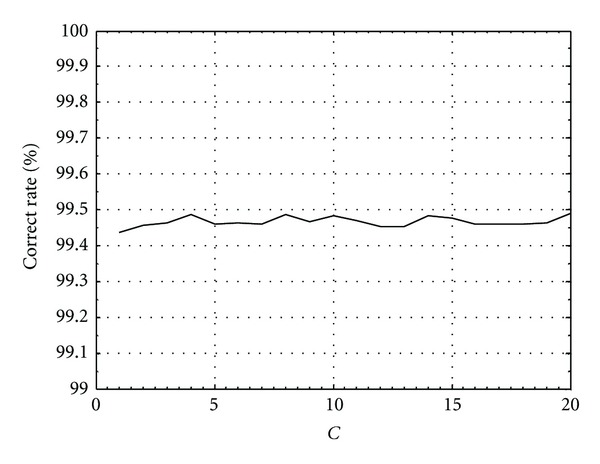
Correct rate of the SVM model with linear kernel for different *C* parameters, SNR = −4 dB, *n* = 5.

To optimize the linear kernel, the only controlling parameter is *C*, which restrains the maximum size of the Lagrangian dual variable. The SVM detector is tested on the 20 sets of 20000 noisy sequences at SNR = 2 dB for various *C* values. The results are shown in Figure 3. While the error performance for various *C* is very similar, it is still ideal to choose a model with the least number of support vector (SV) in order to reduce the complexity. In this case, when *C* is beyond 6, the model gives the same number of SV because variable *α*_*i*_ is no longer constrained by *C*. The correct rate remains around 99.47%, as shown in Figure 4.

**Figure 5:**
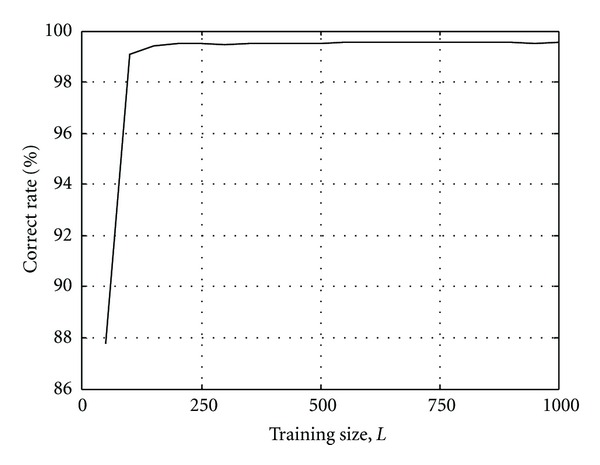
Correct rate of the SVM model with linear kernel for different training sizes, *n* = 5.

**Figure 6:**
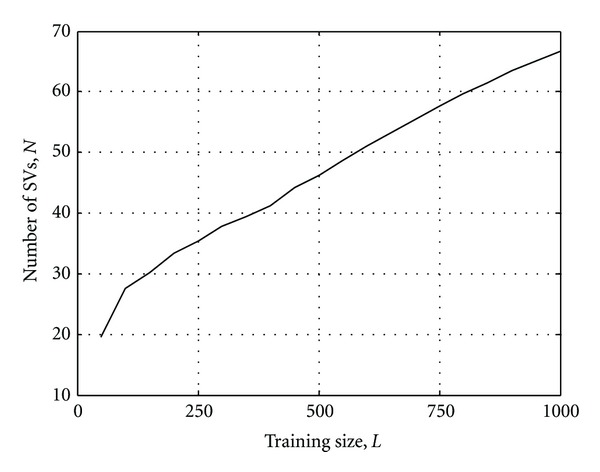
Number of support vectors from the SVM model for different training sizes, *n* = 5.

The training size for the SVM detector is another parameter that the designer needs to control. In general, for any machine learning algorithms, the training size should be as large as possible to improve the prediction of the unknown testing data. The tradeoff in this application is the increased time required to produce and collect the training data. Figures 5 and 6, respectively, show the SVM demodulator's error performance and the number of SVs required on the same system as stated above with different training sizes. When the *C* parameter is fixed at 2, and with a training size of about 200, the performance of the SVM detector would reach to its limit where the increase of SVs would not improve its accuracy.

### 4.3. Posterior Probability Estimates

**Figure 7:**
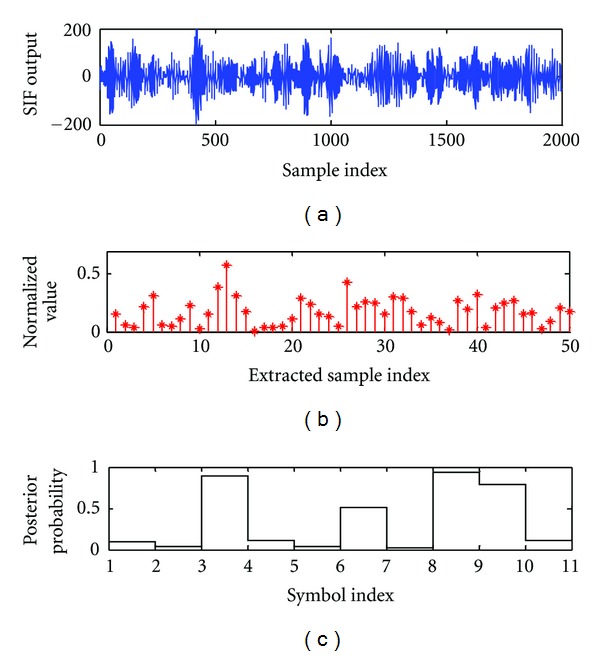
The waveform of SIF output and the posterior probability output obtained by SVM at SNR = −9 dB.

In order to reduce the complexity of the SVM analyzed in Section 2, we select only a few samples from the filter output as the features for training and testing (i.e., *n* = 5 in this case). We depict the probabilities obtained by the SVM output of SNR = −9 dB in Figure 7. The signal in Figure 7 is submerged in noise, so the optimal performance cannot be achieved by using a conventional threshold decision. Yet, the probability which the demodulator output by SVM technique is accurate while a source symbol sequence [0,0, 0,0, 1,1, 0,1, 0,1] is transmitted, and the noise from the part which did not carry any information of the waveform of symbol “1” is almost removed.

**Figure 8:**
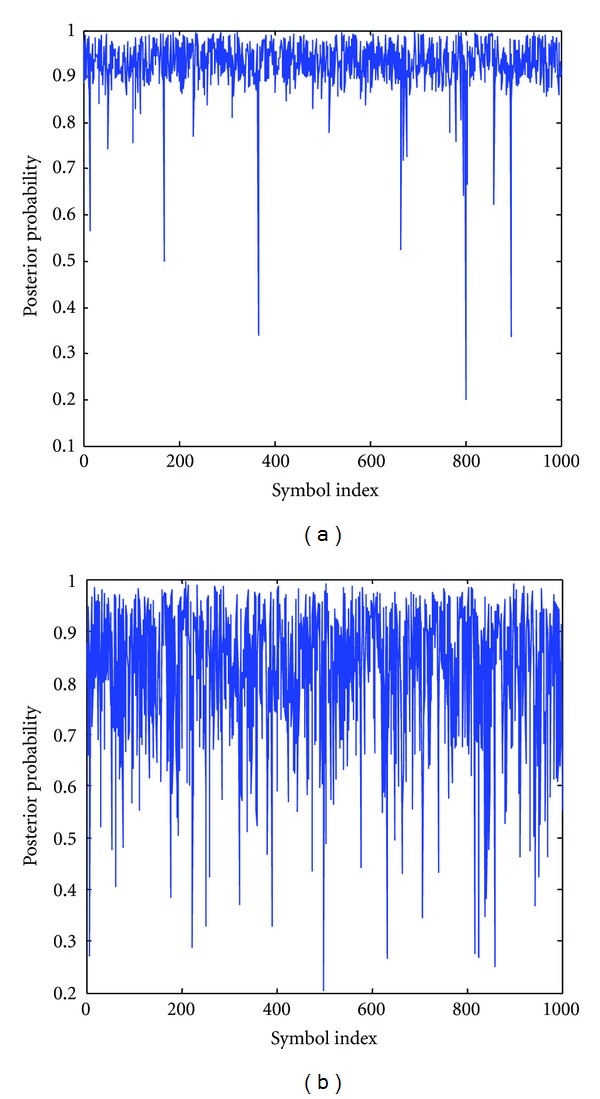
The posterior probability *P*(*y* = 1 | *x*) obtained by SVM and MHY method, in (a) and (b), respectively, where source symbols with all ones are transmitted.

To understand the difference in PPEs, we have plotted the curves for the SVM and the MHY in Figures 8(a) and 8(b), respectively, with SNR = −5 dB. We depict the estimated probabilities *P*(*y* = 1 | *x*) versus the ones when a source symbol sequences with all ones are transmitted. We can appreciate that the SVM PPEs are closer to “1” and less spread, most of the values of demodulation output are between 0.9 and 1. Thereby, SVM estimates are closer to the true posterior probability, which explains its improved performance with respect to the MHY, when we measure the BER after the LDPC decoder.

In a previous subsection, we have shown that the demodulator is based on an SIF and SVM classifier, when we compare performances at a low BER. In this section, we focus on the performance after the sequence has been corrected by an LDPC decoder. The ability of SVM to provide accurate posterior probability predictions boosts the demodulator performance compared to the MHY method.

**Figure 9:**
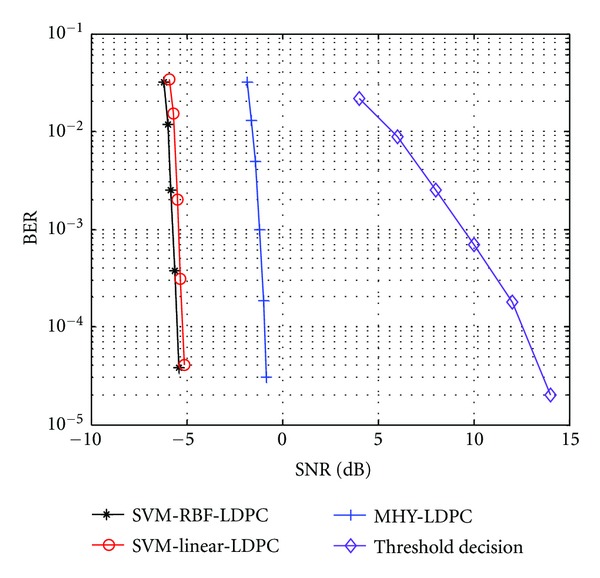
Performance at the output of the LDPC decoder with the soft-input and threshold decision.

**Figure 10:**
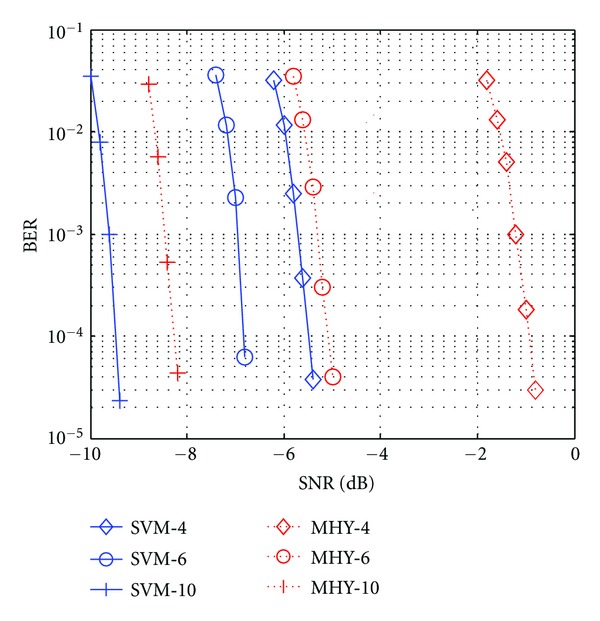
BER performance comparisons of the SVM with MHY method at the output of the LDPC decoder with different sampling rates. Using SVM-4, SVM-6, and SVM-10 for the SVM method (solid lines) and MHY-4, MHY-6, and MHY-10 for the MHY method (dashed lines) with *f*_*s*_ = 4*f*_*c*_, *f*_*s*_ = 6*f*_*c*_, and *f*_*s*_ = 10*f*_*c*_, respectively.

From Figure 8, we can understand that the improved performance of the SVM with respect to the MHY is based on its ability to provide accurate PPEs. In Figure 9, we can appreciate that the SVM-LDPC significantly reduces the BER at lower SNR, because the PPEs are more accurate and the LDPC decoder can rely on these trustworthy predictions. Also, Figure 9 shows that the performance of SVM-RBF-LDPC is a little more superior to SVM-linear-LDPC, it is not the same as the results in Section 4.1 which are analyzed without channel coding. Moreover, the SVM-linear-LDPC decoding outperforms the MHY-LDPC decoding by 4.5 dB and by 18 dB without channel coding when BER = 10^−4^ and sampling rate *f*_*s*_ = 4*f*_*c*_. In Figure 10, we compare the BER performance of the SVM-LDPC with MHY-LDPC by a different sampling rate. Compared to the MHY-LDPC, the SVM-LDPC can upgrade more than 4.6 dB, 1.7 dB, and 1.2 dB for *f*_*s*_ = 4*f*_*c*_, *f*_*s*_ = 6*f*_*c*_, and *f*_*s*_ = 10*f*_*c*_, respectively. This means that the performance of SVM-LDPC improved significantly while the sampling rate is low, and it is not sensitive to the sampling rate for SVM-LDPC. Also, Figure 10 illustrates that it is more superior for the SVM demodulator than MHY in a bad condition.

We have shown that SVM-LDPC is far superior to the MHY method. This result shows that using a method that can predict accurately the PPEs allows the LDPC decoding algorithm to perform to its fullest.

## 5. Conclusions

In this paper, we introduce a nonlinear demodulator which is a novel solution for the EBPSK scheme. We have shown that the performance can be significantly improved by using a linear kernel for demodulation, which has a less computational complexity thus saves the computation time.

SVM is a nonlinear probabilistic classifier that produces accurate PPEs. The performance comparisons of different probabilistic demodulators at the output of an LDPC channel decoder are made, which has shown that the SVM outperforms the MHY with probabilistic output.

The SVM probability output method does not need to estimate the channel noise power *σ*, and uses only a few samples as the features of SVM for training and testing, which reduces the complexity significantly.

A simulator of the system was designed and the BER performance was significantly improved for the SVM-LDPC comparing with the MHY-LDPC approach. Moreover, the SVM method is more robust to sampling rate than MHY method.

Yet, the performance of the system can be improved significantly at the cost of complexity, and the probability is still approximate. More investigations are undertaken to reduce the computational complexity of this approach and test its performance under more severe channel conditions, such as the fading channel.

## Figures and Tables

**Table 1:** Comparison of SVM models.

	Selected kernel	
	RBF	Linear
*C*	4	2
*γ*	8	—
SVs	271	210
